# Prevalence and factors associated with irritable bowel syndrome among medical students in a Malaysian private university: a cross sectional study

**DOI:** 10.11604/pamj.2020.37.151.21716

**Published:** 2020-10-13

**Authors:** Sarvesh Seger, Nur Nabila Binti Nasharuddin, Sabrina Lizy Fernandez, Siti Rahmah Binti Md Yunus, Nicole Teh Mae Shun, Puneet Agarwal, Ismail Burud

**Affiliations:** 1 School of Medicine, International Medical University, Clinical Campus, Seremban, Malaysia

**Keywords:** Irritable bowel syndrome, medical students, depression, anxiety, sleep quality

## Abstract

**Introduction:**

irritable bowel syndrome (IBS) is one of the most common functional gastrointestinal disorder. The medical programme is among the most challenging and stressful undergraduate programmes which may predispose to higher rates of IBS. This study sought to determine the prevalence of undiagnosed IBS and the factors associated with IBS among medical students in a Malaysian private university.

**Methods:**

a cross sectional study was conducted among the medical students from semester 6 to semester 9 (year 3, year 4 and year 5) of a Malaysian private university. The questionnaire consisted of 2 main sections. Section A was on demographic data and section B consisted of the Hospital Anxiety and Depression Scale (HADS), the Pittsburgh Sleep Quality Index (PSQI) and the Rome IV Questionnaire for IBS. Association between the factors gathered and IBS was assessed using the Chi-Square test. Variables with a p-value of less than 0.2 in the univariate analysis were entered into a multivariate analysis model.

**Results:**

number of students who responded were 190 (56.3%) were females, 66.3% were Chinese and 37.9% were from semester 9. Twenty-eight (14.7%) students had symptoms consistent with a diagnosis of IBS. Depression was found to be significantly associated with IBS (adjusted OR: 4.749, CI: 1.809-12.470).

**Conclusion:**

this study suggests that IBS is common among Malaysian medical students. There was a significant association between IBS and depression

## Introduction

Irritable bowel syndrome (IBS) is a presumptive diagnosis defined as a functional bowel disorder in which recurrent abdominal pain is associated with defecation or a change in bowel habits. According to the Rome IV criteria, IBS is characterized by recurrent abdominal pain, on an average of at least 1 day a week in the last 3 months, associated with two or more of the following criteria: related to defecation, association with a change in frequency of stool or association with a change in form (appearance) of stool. This criterion has to be fulfilled for the last 3 months with symptom onset at least 6 months before diagnosis [1]. The presentation of IBS varies across individuals and are subdivided according to the Rome IV classification into four bowel patterns; which are IBS-D (diarrhea predominant), IBS-C (constipation predominant), IBS-M (mixed diarrhea and constipation) and IBS-U (unclassified as it cannot be categorized into the other 3 subtypes) [1]. A diagnosis of IBS is considered after ruling out organic causes of gastrointestinal symptoms.

The estimated prevalence of IBS is 10% to 25% in different regions worldwide, making it the most common functional gastrointestinal disease [2]. The prevalence of IBS among medical students in Malaysia was found to be 15.8% [3]. This is higher than the prevalence of IBS in the Malaysian general population, which was found to be 14% [4]. The worldwide prevalence of IBS among medical students ranged from 9.3 to 35.3% [5], which is also higher than the worldwide general population. Studies related to IBS focused on the general population and those on medical students were mainly outside Malaysia. When compared to other countries, the climate of Malaysia is different, and the population has a diverse culture with people having different dietary habits. Medical students differ from the general population in terms of the high academic and professional standards placed on them. Despite having symptoms few patients seek medical care [6]. Currently, there is no cure for IBS; hence treatment aims to help manage the symptoms. Initial therapy for mild or moderate IBS would include dietary modifications [7]. There is a relative paucity of data from Asian countries, which gives the impression that IBS is not common in this population [3]. Several studies have demonstrated IBS is more prevalent among females. Anxiety and depression were also found to be associated with IBS [4, 8]. Poor sleep quality was found to be associated with IBS as well [9-11]. The aim of this study is to determine the prevalence of IBS among medical students in a Malaysian private university and the factors associated with IBS in this group.

## Methods

**Study design, setting and participants**: this cross-sectional study was carried out on medical students from Semester 6 to Semester 9 of a Malaysian private university from August 2018 to December 2018. This study was conducted with approval from the International Medical University Joint-Committee on Research and Ethics (IMU Research Project Number: CSc/Sem6(09)2019). The total population of students was 372. The sample size was calculated by Raosoft Sample Size Calculator for this study according to 95% confidence interval was 190. Students previously diagnosed with IBS were excluded from the study.

**Study variables:** independent variables that were evaluated to detect the association with IBS were: ethnicity, gender, year of study, anxiety, depression, and sleep quality.

**Study instrument:** a self-administered questionnaire in English which had two sections was developed. Section A consisted of demographic data which included gender, race, age and semester. Section B consisted of the Rome IV questionnaire which screens for IBS, Hospital Anxiety and Depression Scale (HADS) questionnaire which screens for anxiety and depression and the Pittsburgh Sleep Quality Index (PSQI) screens for poor sleep quality [12-14]. The necessary permission was taken to use these questionnaires in our study.

**Data collection:** the English language was used in the questionnaires to maintain clarity and uniformity. The questionnaire was pre-tested among 10 students before actual data collection. The pre-tested data was discarded after preliminary assessment and never included in the final analysis. Data collection was carried out by approaching participants using convenience sampling method. The participants were approached by going from class-to-class of the respective semesters. The participants were briefed about the study in English. A study information sheet was provided, and written consent was obtained before administering the questionnaire. We notified the subjects who screened positive and suggested they seek medical attention for their condition.

**Statistical analysis:** the data collected were tabulated and analyzed by using the Statistical Package for Social Sciences (SPSS) version 26.0. Frequency tables and cross-tabulations were generated. Chi-square test was applied for comparison of categorical variables. Odds ratio and its 95% confidence interval were used to study the strength of associations of categorical variables. Variables with a p-value of less than 0.2 in the univariate analysis were included into a logistic regression model. Multivariate analysis using binary logistic regression was used to study the independent effect of each variable over the outcome. The results of the regression analysis were reported using adjusted odds ratio and a 95% confidence interval.

## Results

**General characteristics of participants:** of the 190 students, 72 (37.9%) were from Semester 9, 78 (41.1%) were 23 years old, 107 (56.3%) were female and 126 (66.3%) were Chinese. Their age ranged from 21 to 27 years old with a mean of 23.18. The demographic characteristics of the respondents are mentioned in Table 1 and Table 2.

**Table 1:** demographics and risk factors of participants with and without IBS

	Non-IBS	IBS	Total
	n (%)	n (%)	n (%)
Total	162 (85.3)	28 (14.7)	190 (100)
**Gender**			
Male	69 (83.1)	14 (16.9)	83 (43.7)
Female	93 (86.9)	14 (13.1)	107 (56.3)
**Ethnicity**			
Chinese	107 (84.9)	19 (15.1)	126 (66.3)
Malay	19 (90.5)	2 (9.5)	21 (11.0)
Indian	26 (89.7)	3 (10.3)	29 (15.3)
Others	10 (71.4)	4 (28.6)	14 (7.4)
Depression^*^	28 (73.7)	10 (26.3)	38 (20)
Anxiety^*^	63 (80.8)	15 (19.2)	78 (58.9)
**Poor sleep quality^#^**	81 (82.7)	17 (17.2)	98 (51.6)
**Semester**			
6	33 (86.8)	5 (13.2)	38 (20.0)
7	58 (84.1)	11 (15.9)	69 (36.3)
8	11 (100)	0 (0)	11 (5.8)
9	60 (83.3)	12 (16.7)	72 (37.9)

* The screening for depression and anxiety was based on the Hospital Anxiety and Depression Scale score of > 7. ^#^ Screening for poor sleep quality was based on the Pittsburg Sleep Quality Index

**Table 2:** univariate and multivariate analysis of risk factors and demographic features associated with irritable bowel syndrome

	Unadjusted OR (95% CI)	p-value	Adjusted OR (95% CI)	p-value
**Depression**	**4.749 (2.017-11.182)**	**0.000**	**4.749 (1.809-12.470)**	**0.002**
**Anxiety**	1.813 (0.809-4.064)	0.145	1.000 (0.390-2.561)	1.000
**Gender**	0.742 (0.332-1.657)	0.466	-	
**Ethnicity** (Chinese/non-Chinese)	0.922 (0.703-1.652)	0.852	-	
**Poor sleep quality**	1.545 (0.682-3.504)	0.295	-	
**Year of study** (Fourth/fifth Year)	0.961 (0.565-2.876)	0.558	-	

**Prevalence of irritable bowel syndrome:** of the 190 medical students recruited in this study, 28 (14.7%) fulfilled the criteria for having IBS.

**Correlates of irritable bowel syndrome:** univariate and multivariate analysis showed that IBS was associated with depression. Among the 38 respondents who were found to have depression, 13 (34.2%) were reported to have IBS. In comparison, only 9.9% respondents who did not have depression were reported to have IBS. This correlation was statistically significant as shown in the univariate analysis (unadjusted OR = 4.749, CI = 2.017-11.182) and multivariate logistic regression analysis (adjusted OR = 4.749, CI = 1.809 - 12.470). Prevalence of anxiety and sleep quality showed that 58.9% of the respondents had positive symptoms of anxiety, while 51.6% reported poor quality of sleep. Twenty percent (20%) of the respondents in this study also showed possible depression.

Based on the HADS questionnaire, 78 students were found to have possible anxiety and among them 15 (19.2%) have possible IBS. However, this is not statistically significant in both univariate (unadjusted OR = 1.813, CI = 0.809 - 4.064) and multivariate analysis (adjusted OR = 1.000, CI = 0.390 - 2.561). With regards to the Pittsburg Sleep Quality Index (PSQI), 98 students were found to have poor sleep quality and among them 17 (17.3%) have possible IBS. However, this is not statistically significant (OR = 1.545, CI = 0.682-3.504). A higher percentage of males (16.9%) were found to have possible IBS compared to females (13.1%). However, the difference was not statistically significant (OR = 0.742, CI = 0.332 - 1.657). A greater percentage of Chinese were found to have possible IBS (15.1%) compared to non-Chinese (14.1%). However, the results were not statistically significant (OR 0.992, CI = 0.391 - 2.172). We found 12 (16.7%) students in their final year of study had possible IBS compared to 16 (13.6%) students who are not in their final year of study. However, this is not statistically significant (OR = 1.275, CI = 0.565-2.876).

## Discussion

The prevalence of IBS among the medical students in this study was 14.7%. This is slightly lower than another prevalence study which found that 15.8% of medical students in Malaysia had IBS [3]. There is a higher prevalence of IBS among medical students compared to the general population in Malaysia, which is 14% [4]. The worldwide prevalence of IBS among medical students is 9.3-35.3% [5]. The results of our research also fall within this range. A possible reason for the higher prevalence of IBS among medical students is due to higher stress levels in this population as a result of the rigorous academic and clinical training.

The prevalence of IBS in the general population is 4.4% in Thailand [15], 2.3% in Singapore [16] and 6.6% in Hong Kong [17]. The prevalence of IBS in Asia ranges from 3.2% to 22.8% [18], while the prevalence in Europe and North America, ranges from 10-15% [19]. The prevalence in Malaysia is higher than most of Asia, but lower than the West [18]. A possible rationale behind this phenomenon might be related to the different dietary content, and socioeconomic conditions in Malaysia compared to the West as well as the rest of Asia. Depression has a significant association with IBS in this study (odds ratio = 4.749, confidence interval = 2.017-11.182). A 2003 study among medical students in Malaysia reported a significant association between depression and IBS [3]. The exact mechanism is not clear, but it has been postulated that alteration in central nervous system (CNS) responses to psychological and physical stressors lead to colonic spasms, which results in the manifestation of IBS symptoms [20]. There is no pharmacologic cure for IBS, hence managing depression in the individual may ameliorate the severity of IBS.

More males (16.9%) were found to have possible IBS compared to females (13.1%). However, the results were not statistically significant A Korean study involving 319 sixth-year medical students found that the prevalence of IBS among males and females was 41% and 25%, respectively [21]. Furthermore, a study in Pakistan stated that males are more likely to report IBS symptoms compared to female students [9]. However, other studies conducted in Malaysia and Pakistan showed that the reverse was true [3, 22, 23]. Studies indicating a higher prevalence of the disease among males referred to the cultural barrier as the factor that can limit female students from reporting the disease, whereas studies with higher female predilection related the disease to their health care-seeking behavior and possible association with the menstrual cycle [22]. In terms of ethnicity, a greater percentage of Chinese were found to have possible IBS (17.7%) compared to non-Chinese (16.3%). However, the results were not statistically significant. This concurs with another study in which Malaysia´s multi-ethnic population did not show significant ethnic differences in preponderance of IBS [4].

In terms of year of study, a greater percentage of final year students were found to have possible IBS (16.7%) compared to non-final year students (13.6%). However, the results were not statistically significant. This correlates with a study performed among medical students in Mongolia that found students in the later stages of their study were more likely to develop IBS [8]. A systematic review done among medical students from different countries also showed higher prevalence of IBS among students in the higher level of studies [5]. This may be the result of the increased workload as well as clinical and academic demands encountered during the more senior years of medical education.

This study portrays a higher risk of IBS among students with poor sleep quality, however it was not statistically significant. A study among medical students and interns in Saudi Arabia showed that students who slept less than eight hours per day had a higher prevalence of IBS [9]. Sleep disturbances are known to have profound physiologic consequences, including increases in pro-inflammatory cytokines and cortisol levels, while at the same time diminishing parasympathetic tone [24]. Those who have anxiety were found to have a higher risk of IBS, but the results were not statistically significant. The most prevalent psychiatric comorbidity in patients who are diagnosed with functional gastrointestinal disorders is anxiety disorder. The role of psychosocial modifiers of IBS was accentuated in the “multi-dimensional clinical profile” which was presented by the Rome IV criteria [25]. This is because psychological factors do play a role in influencing IBS symptoms which essentially lead to poor outcomes. The symptoms of IBS worsen the quality of life of medical students; hence, the associated risk factors should be taken note of. Although there is no known cure for IBS, there are existing preventive measures of the predisposing factors. These include raising awareness of the society, dietary adjustments as well as various psychological and psychiatric interventions.

Ideally there should be more studies carried out to conclude the exact prevalence among medical students on a broader scale and viewing the impact on the student´s quality of life. The burden of IBS on the healthcare system can be substantial, eventually leading to a socioeconomic burden of the disease on the people who suffer from IBS. Primary health care centers in Malaysia should reach out by providing educational campaigns and sessions for medical students with the aim of shedding light onto different methods of disease control and at the same time encourage medical students to reach out if they develop IBS symptoms. Through this approach, early detection can be carried out and play a pivotal role in the management of IBS. As depression has a significant association with IBS a clinician should have a high index of suspicion for depressive symptoms, evaluate the patient´s mental health status and consider the possibility of IBS when treating medical students.

## Conclusion

In conclusion, the prevalence of IBS amongst medical students in the Malaysian private university was higher compared to the Malaysian population. Depression was found to be significantly associated with IBS among medical students.

### What is known about this topic

- The prevalence of IBS is higher among medical students compared to the general population;
- Female gender, poor sleep quality, anxiety and depression are associated with IBS.

### What this study adds

- Depression is associated with IBS among medical students in Malaysia;
- The prevalence of IBS is higher among medical students in Malaysia compared to other Asian countries but lower than western countries.
